# Recognizing and Mitigating Canine Stress in Human–Canine Interaction Research: Proposed Guidelines

**DOI:** 10.3390/ani15111665

**Published:** 2025-06-05

**Authors:** Simone B. Sidel, Jaci Gandenberger, Kerry Murphy, Kevin N. Morris

**Affiliations:** 1 Animal Care Center of Castle Pines, 562 Castle Pines Pkwy C5, Castle Rock, CO 80108, USA; drsimonesidel@gmail.com; 2 Institute for Human-Animal Connection, University of Denver, 2148 S High St., Denver, CO 80208, USA; jaci.gandenberger@du.edu (J.G.); kerrymurphy10@gmail.com (K.M.)

**Keywords:** human–canine interactions, human–animal interactions, canine welfare, Fear Free, FAS spectrum, canine salivary cortisol, canine low-stress handling, canine stress markers

## Abstract

The research into human–canine interactions (HCIs) has grown substantially, yet limited attention has focused on the welfare of canines involved, particularly pet dogs owned by volunteer participants. To address this gap, we conducted a secondary analysis of data from a randomized controlled trial, examining canine welfare during an acute human stress protocol. Our methodology incorporated evidence-based screening tools, environmental modifications, researchers trained in canine behavior assessments and safe interactions, and canine stress monitoring using the Fear Free™ Canine Fear, Anxiety, and Stress (FAS) Spectrum. Dogs’ stress levels showed a non-significant increase from the rest to stressor phase (0.80 to 1.00, *p* = 0.073) and a significant decrease during recovery (1.00 to 0.48, *p* < 0.001). Only two dogs (7.6%) required withdrawal due to elevated stress levels, though these levels remained within acceptable safety parameters. The peak stress remained within acceptable limits, with only 24% (6 of 25) reaching an FAS score of two during the TSST. By final recovery, 96% of dogs achieved FAS scores of zero to one (Green Zone), indicating relaxed states. Salivary collection proved challenging, highlighting limitations in low-invasive physiological measurement techniques. Based on our findings and literature review, we propose standardized guidelines for HCI research, including thorough pre-screening, environmental preparation, researcher training, stress-monitoring protocols, and informed consent procedures emphasizing withdrawal rights. These guidelines aim to establish ethical standards for this rapidly expanding field, protecting canine participant welfare while enabling valuable research to continue.

## Simple Summary

While studies examining the benefits of human–canine interactions have increased dramatically, little attention has focused on the welfare of dogs in these studies. Currently, no guidelines exist for ensuring the welfare of pet dogs in human-focused HCI research. We developed and tested methods to protect dogs during a psychological stress test where human participants underwent a challenging task while their pet dog was present. We carefully screened dogs beforehand, created a comfortable environment with beds and water, and monitored stress signs throughout the study. Using a scale that measures fear and anxiety through body language, we found that dogs’ stress levels remained mostly low, with only two dogs needing to be withdrawn. Our proposed guidelines include proper screening, environment preparation, researcher training, and stress-monitoring protocols. These recommendations will help researchers conduct more ethical studies that respect dogs as true participants rather than research tools, ensuring their welfare while still allowing valuable research that benefits both species to continue.

## 1. Introduction

The research into the benefits of human–canine interactions (HCIs) has grown dramatically in recent years, covering topics ranging from canines’ potential roles in improving symptoms of stress and trauma [1], supporting recovery from substance use disorders [2], managing symptoms of autism [3], enhancing engagement in mental health treatment [4], and more. While HCIs are a species-specific subset of Human–Animal Interactions (HAIs), it is important to clearly differentiate this work from Animal-Assisted Interventions (AAIs). Pet Partners (previously the Delta Society) clarifies that an HAI “includes, but is not limited to, emotional, psychological, and physical interactions of people, animals, and the environment”, whereas AAIs “are goal-oriented and structured interventions that intentionally incorporate animals in health, education, and human service for the purpose of therapeutic gains and improved health and wellness” [5]. Additionally, AAIs include Animal-Assisted Therapy (AAT), Animal-Assisted Education (AAE), and Animal-Assisted Activities (AAAs) [5]. The fundamental difference is that HAIs encompass organic, everyday interactions between humans and animals, whereas AAIs are structured, goal-oriented interventions designed for specific therapeutic outcomes. While there is extensive literature on the importance of animal welfare and stress mitigation guidance in AAI work [6,7,8,9,10,11,12,13], there are limited studies on canine welfare and stress reduction methodologies in pet dogs owned by volunteer participants in HCI studies.

Within the United States, Institutional Review Boards (IRBs) are responsible for safeguarding human subjects’ rights, privacy, and welfare, while Institutional Animal Care and Use Committees (IACUCs) oversee the use of animals in research. However, because many HCI studies are focused on the impacts on the human participants, typically do not include university-owned animals, or are considered “non-invasive” by IACUC standards, they often do not fall under IACUC oversight. That said, volunteer participant pet dogs in HCI research are often exposed to numerous stressors, many of which may be easily overlooked by researchers if they are not properly educated on how canines experience their environment, how canines communicate emotions and manage stress, and how canines read and respond to human body language and actions. In addition, canine welfare can further be amplified by understanding canine comfort needs. Some common canine stressors that can be found in HCI study designs include loud noises, aversive scents, fear pheromones from other dogs, separation from their guardian (even if temporarily), researchers undereducated in low-stress canine approaches and canine communication techniques, and researchers undereducated in reading and responding to canine body language. Many of these known stressors can be easily recognized, accounted for, and mitigated in a well-thought-out and planned study design.

While the 2010 Animal Research: Reporting of In Vivo Experiments (ARRIVE) guidelines and their 2020 update partially fill the gap in the guidance for HCI researchers, their primary focus is not on animal welfare; instead, it is to ensure research studies are reported in enough detail to fill knowledge gaps and promote replicable research [14]. Although guidelines have been created to prioritize canine welfare in other similar utilizations, such as the Lincoln Education Assistance with Dogs (LEAD) Risk Assessment Toolkit for dogs involved in AAIs [6] or veterinary clinic studies committees (VCSTs) for non-federally funded research involving client-owned dogs [15], no such guidelines exist for non-university-owned dogs in human-focused HCI studies.

Without a clear source of guidance or oversight, there is a high risk of dogs being subjected to stressful interactions or researchers overlooking the impact of study environments and protocols on canine welfare. In extreme situations, there is also a risk that particularly distressed dogs may become aggressive or dangerous to those around them. Thus, there is a need to develop a framework that can be used widely by HCI researchers to help ensure responsible practices that protect the welfare of all participants, including the canine participants, and to establish ethical standards for this rapidly expanding field.

Towards that end, we conducted a secondary analysis of data from a randomized controlled trial examining the effects of the presence of participant pet dogs on human acute stress responses during the Trier Social Stress Test [16]. While the primary study focused on human stress outcomes, the study design incorporated a particular focus on canine welfare considerations and methodological approaches, as well as the data collection of the pet dog stress levels throughout the study. Drawing from this experiment and the existing literature, we present our proposed guidelines to prioritize canine welfare in HCI research utilizing volunteer participant pet dogs.

## 2. Materials and Methods

### 2.1. Study Design and Secondary Analysis Approach

This study represents a secondary analysis of data from a randomized controlled trial that examined the effects of the presence of a pet dog on human acute stress responses during the Trier Social Stress Test (TSST) [16]. In the primary study, participants were randomized to undergo the TSST either with their pet dog present (experimental group) or without their pet dog present (control group). While the primary study focused on human physiological and psychological stress outcomes, the present analysis examines the canine welfare considerations, methodological approaches for canine stress mitigation, and the systemic monitoring of canine stress responses throughout the primary experiment. The present secondary analysis focuses exclusively on data from the 27 participants who were randomized into the dog group, as only these participants had canine welfare data collected. For comprehensive methods and results regarding the human participants, see Gandenberger et al., 2024 [16].

### 2.2. Study Participants and Recruitment

The primary study recruited participants through multiple channels to ensure a wide and diverse reach of guardian–dog dyads, including PetSmart dog training classes, the research facility IACUC network, recruitment flyers around apartments in the surrounding metro area, recruitment flyers on the campus of the research facility, and Denver Pet Partners, among others.

Initially, we considered limiting participants to those who had a trained therapy or service dog or to those whose dogs had passed a Canine Good Citizen (CGC) test [17]. However, we felt that this would significantly reduce the generalizability of our findings and potentially also restrict our ability to recruit enough participants. Therefore, all dogs in the study were pet companion dogs and not dogs trained or otherwise involved in any service or therapy. We next discussed requiring all participants to meet with a canine behavior consultant who could assess their dogs’ suitability for the study, but due to budget and participant burden considerations this proved unfeasible.

All potential participants were the guardians (also known as “owners”) of the dogs and thus had long-standing relationships and bonds with the dogs. The participants were required to complete a screening questionnaire to assess their dogs’ suitability for the study conditions (detailed in Section 2.5). Data collection took place between September 2021 and February 2022. For comprehensive demographic and procedural details regarding the human participants, see Gandenberger et al., 2024 [16].

### 2.3. Canine Welfare Protocol Development and Researcher Training

We designed the canine-focused elements of our study protocol with the input of three individuals: a dual-certified canine behavior consultant, a researcher who had received a certification through Fear Free™ [18], and a researcher with experience conducting canine stress assessments for past research. The canine behavior consultant held both Certified Professional Dog Trainer, Knowledge-Assessed (CPDT-KA) and Certified Dog Behavior Consultant (CDBC) credentials. CDBC credentials are provided through the International Association of Animal Behavior Consultants (IAABC). These certifications require extensive documented experience (300–500 h), comprehensive examinations, case studies, and professional references [19]. The Fear Free-certified researcher completed the Animal Trainer Certification Program, which specifically focuses on preventing and alleviating fear, anxiety, and stress in pets through evidence-based practices [20]. We selected the Fear Free Animal Trainer Certification Program as it was the most relevant to reducing stress in dogs involved in HCI research. This certification provided our facilitators with training in identifying fearful, anxious, or stressed body language; techniques for calmly transitioning animals between environments; and creating spaces sensitive to canine needs [21]. The third researcher contributed valuable practical experience in assessing canine stress responses in previous research protocols.

As a team, these individuals utilized their personal experience and expertise to identify study conditions that had the potential to cause distress to a dog and developed a screening questionnaire for potential participants (detailed in Section 2.5).

### 2.4. Study Environment Preparation

Before any TSST experiments were conducted, the research team walked through the study facility and assessed the area with an eye towards canine comfort and safety to identify and mitigate potential stressors for participating dogs. Environmental factors known to increase canine stress include loud noises, cleaning chemical odors, pheromones from stressed animals, slippery surfaces, bright lights, and guardian separation [8,21,22,23,24,25,26].

Wherever possible, adjustments were made to the environment to enhance canine welfare. These modifications included outfitting the rest/recovery room with a dog bed, full water bowl, and extra water for refills [6,22,25,26,27]. The rest/recovery room already provided good traction with carpeting, and an air filter with an activated carbon filter (used to absorb odor) was added to minimize the scent of previous dogs, as well as to provide white noise and reduce potentially aversive sounds [25,26]. Guidelines for dogs in veterinary settings and animal-assisted interventions consistently emphasize the importance of hydration access [6,22,25,26,27]. Similarly, LEAD welfare tools highlight the necessity of providing a designated rest area with a blanket or bed, away from human interaction [6].

Additional items were made available in the TSST room, including a pee pad and an optional yoga mat to provide a non-slip surface for dogs who struggled with tile floors [25,26]. Each experiment was conducted with at least 24 h between participants with the air filter running continuously between sessions, with an aim to decrease the likelihood of lingering smells or pheromones from previous dogs or humans [25,26].

Some factors were out of the control of the study facilitators, such as the wattage of the lights in the study room. However, it is important to note that dogs have a tapetum lucidum, which allows them to see light in greater abundance and makes what humans perceive as soft lighting seem brighter and aversive [21]. Therefore, whenever possible, more dull lighting, particularly 60 W lightbulbs, is preferred to fluorescent bulbs [21].

### 2.5. Participant and Dog Screening Questionnaire

Before being accepted into the study, all potential participants were required to complete a screening questionnaire created by the expert team who were described in Section 2.3 (Supplemental Figure S1). The questionnaire assessed their dogs’ suitability for specific study conditions, including whether the dog would be comfortable taking an elevator to reach the fifth floor, being around several unknown adults, having their leash held by an unknown adult during blood draws, being in a laboratory environment, remaining calm while their guardian was temporarily distressed, and being leashed at all times except when alone with their owner.

Answering “no” to any yes/no question was a disqualifier, while answering “maybe” prompted a follow-up conversation with the research team to gather more information and issue a collaborative determination about whether the dog was a good fit for the study.

Potential participants were also asked about their dog training experience and ability to recognize canine stress signals, including specific signs their own dog displayed when stressed. These questions served dual purposes. First, they helped the research team assess the reliability of participants’ responses, since pet guardians with training experience typically demonstrate a better understanding of dog behavior [12] and are more accurate at identifying stress in dogs. Second, they provided individualized insights into each dog’s stress signals, which could be taken into account by the study facilitators during the experiment. This is important because canine expressions of stress can be unique to each individual [12]. Additionally, participants were asked if their dog had ever shown aggression to other people or dogs, as this would be a disqualifier due to the nature of the study.

A member of the research team reviewed each set of responses, asked follow-up questions as needed, and was available to answer any questions from potential participants. Potential participants who were unable to confidently answer any of the questions spoke with the study facilitator to obtain further clarity and mutually determine whether their dog was likely to become excessively distressed during the experiment. Participants understood that they would be undergoing a stressful situation to investigate whether the presence of their dog impacted their stress response, but they did not know the specific details of what they would experience.

### 2.6. Experimental Protocols and Procedures

The experiment consisted of three phases: a 30 min rest period in a quiet room (rest/recovery room), a 15 min human acute stress induction involving public speaking and mental arithmetic tasks before a panel of evaluators wearing white lab coats in a separate room (TSST room), and a 45 min recovery period in the rest/recovery room. At the end of each phase, participants completed standardized questionnaires, provided saliva samples, and underwent blood collection by a trained phlebotomist. Participants in the dog group were instructed to collect saliva samples from their dogs at the end of each phase (detailed in Section 2.8). The pet dogs remained under the control of the participant at all times during the study, except for while the participant underwent blood collection (described in more detail below). For more comprehensive methods and results regarding the human participants, see Gandenberger et al., 2024 [16].

Each experiment was managed by a study facilitator who had received a Fear Free certification. While the Fear Free certification program was the most relevant available training, it is primarily designed for veterinary staff, pet professionals, and pet guardians rather than researchers, suggesting a need for specialized HCI researcher training programs. Participants were instructed to bring their dogs on 6-foot non-retractable leashes for safety. Upon arrival, participants and their dogs were greeted by study facilitators who utilized their body language to avoid appearing threatening and to minimize the stress experienced by the dog [21,22,24,26,28,29]. This allowed dogs time to develop an initial comfort with the facilitator. Before entering the facility, dogs were allowed the opportunity to relieve themselves outdoors, preventing potential restlessness during the 90 min experiment session. When the participant and dog were ready, the facilitator guided them inside. Upon entry into the rest/recovery room, dogs were given time to sniff and explore the room on leash before the participants were verbally guided through the consent procedure [22].

Once the participant and dog were settled in the rest/recovery room, each participant was verbally guided through and signed an informed consent document. This study was approved by the University of Denver’s Institutional Review Board (IRB protocol number 1664556) and Institutional Biosafety Committee (IBC protocol 1773388), and all methods were performed in accordance with their guidelines and regulations. Because the participating dogs were not owned by the university, Institutional Animal Care and Use Committee (IACUC) approval was not required. Each dog’s legal guardian provided permission for their participation in the study. All participants provided written informed consent.

Once informed consent was completed, participants were asked to document data about their dogs, including age, sex, spay/neuter status, breed, and length of ownership (Table 1). Once all intake tasks were completed, the participant and dog were left alone in the rest/recovery room with a closed door containing a window for a 30 min rest phase. During this and a similar recovery phase near the end of each experiment, participants were allowed to let their dog off-leash. Participants were not given any other instructions on how to interact with their dog so that interactions could be as natural as possible. This was the only time during the experiment that dogs were allowed off-leash.

During the TSST phase of the experiment, the dogs were kept on a 6-foot non-retractable leash with the participant for their safety and that of the people around them. During participant blood draws, the study facilitator assumed control of the leash for participant and phlebotomist safety, keeping the dog in sight but outside of the reach of the participant to ensure the safety of all involved in the experiment. At all other times, the dogs remained under the participants’ control. Participants were otherwise encouraged to interact naturally with their dogs.

Throughout the experiment, the dogs remained in sight of the study facilitator except during designated rest/recovery phases. This allowed for the continued assessment of canine body language and intervention if signs of excessive distress emerged. See Section 2.7 for more detailed information regarding the assessment of canine body language.

### 2.7. Canine Stress Monitoring and Observational Data Collection

Visual signs of the dogs’ emotional state were monitored using the Fear Free Canine Spectrum Fear, Anxiety, and Stress (FAS) scale. The FAS score is based on a 5-point scale to assess emotional arousal based on canine body language [18]. In the Green Zone (FAS score 0 to 1), dogs appear relaxed, excited, or mildly anxious, and interaction is generally safe. Behaviors include a relaxed posture, soft eyes, a neutral or slightly open mouth, neutral/forward ears, and a neutral/high tail carriage. In the Yellow Zone (FAS score 2 to 3), dogs show moderate fear, anxiety, or stress, requiring a slow and considerate approach. Behaviors include panting with a tight mouth, ears back/to the side, a lowered (not tucked) tail, fidgeting, head turning away, and hesitancy to interact. In the Red Zone (FAS score 4 to 5), dogs enter into a fight/flight/freeze response, requiring an immediate cessation of the interaction. Signs include a fully tucked tail, trembling, a hunched posture, piloerection, escape attempts, snarling, or lunging. Refer to https://fearfreepets.com/fas-spectrum/ (accessed on 11 April 2025) for more detailed descriptions and images [18].

The facilitator recorded FAS scores at three timepoints: at the end of the rest phase, during the TSST phase (specifically when participants were presenting to the panel of strangers), and at the end of the recovery phase. For the rest and recovery FAS score assessment, the facilitator carefully observed through a window into the rest/recovery room, taking precautions to remain undetected by the dogs to prevent influencing their behavior during the assessment.

Any dog that exhibited an FAS score of 4 or 5 would be pulled from the experiment immediately to prioritize welfare and safety. Any dog that exhibited an FAS score of 3 during the rest or recovery phases was given extended time until returning to an FAS score of 2 or below, ensuring their comfort before proceeding. However, an FAS score of 3 during the TSST portion necessitated exclusion from the study, as interrupting this standardized procedure would compromise data validity.

### 2.8. Canine Salivary Cortisol Sample Collection

At the end of each phase (rest, TSST, recovery), participants were asked to collect saliva samples from their dogs using an oral swab. A training video on how to properly use the collection swabs was provided to participants during the experiment sign-up process. The study facilitator oversaw the salivary swab collection, and if dogs’ FAS score escalated to a 2 or above (repeatedly turning head away, avoidance of handling, fidgeting, freezing, growling, lip lifting, etc.) for longer than 3 s, attempts at salivary collection were immediately stopped.

### 2.9. Statistical Analysis

We conducted a repeated measures ANOVA to analyze changes in the dogs’ observed stress levels, as recorded at the end of the rest phase (rest), during the TSST (TSST), and at the end of the recovery phase (recovery). This analysis specifically examined canine welfare outcomes and stress response patterns, representing a distinct analytical focus from the primary study’s emphasis on human physiological responses. The canine stress analysis utilized FAS scores from all dogs for which complete observational data were available, regardless of their accompanying guardian’s data completeness in the primary study.

While human stress data were collected simultaneously in the primary study, the present analysis focuses exclusively on canine welfare outcomes. Correlation analyses between human and canine stress responses were beyond the scope of this secondary analysis but represent an important avenue for future research.

## 3. Results

Of the 111 individuals who expressed interest in this study, 55 (49.5%) participated. Among those who did not participate, 27 (48.2%) did not respond to the email that provided study details and initial screening questions. Six (10.7%) others did not respond when asked to schedule a time for the consent procedure. Of those who shared why they were not participating, the most common reason was schedule conflicts (12, 21.4%), followed by their dog not fitting the screening requirements (7, 12.5%). One person (1.8%) withdrew after being informed that they had been randomized to participate without their dog.

Among the 55 participants, 27 were randomized into the experimental (dog) group, with 26 successfully progressing to informed consent. See Table 1 for detailed information about the dogs’ demographics. For comprehensive demographic details regarding the human participants, see Gandenberger et al., 2024 [16].

None of the canine participants exhibited behaviors consistent with an FAS score of four or five (Red Zone), and only two (7.6%) exhibited behaviors consistent with an FAS score of three (Yellow Zone), requiring withdrawal from the study. In one case, this occurred prior to the informed consent, during which the dog was unable to settle (but was not attempting to flee or freeze and was still intermittently interacting with the guardian) after 15 min, indicating an FAS score of three, and so the participant withdrew before starting the experiment (and thus this score was not in the final data set of 26 dogs). The second case occurred during the participant’s first blood draw. The dog, who weighed over 100 pounds, pulled strongly towards the participant (but did not have a tucked tail, hunched body, tension, or aggression), indicating an FAS score of three. An FAS score of three is still in the Yellow Zone, indicating to proceed slowly and considerately, but is not yet in the Red Zone, which requires the immediate cessation of the interaction. However, because of the dog’s size and level of distress, the study facilitator was unable to keep the dog a safe distance from the participant and phlebotomist. Out of concern for everyone’s safety during the blood draw, that pair was withdrawn from the study. No dogs required additional time in the rest or recovery phases to de-escalate their FAS score. Two other participant–dog pairs were excluded from the final data set due to the inability to collect blood samples from the participants. We obtained full data from 22 human–canine dyads, and full canine data from 25 dogs. All canine data were analyzed for this study, even if their accompanying guardian had incomplete data.

**Figure 1:**
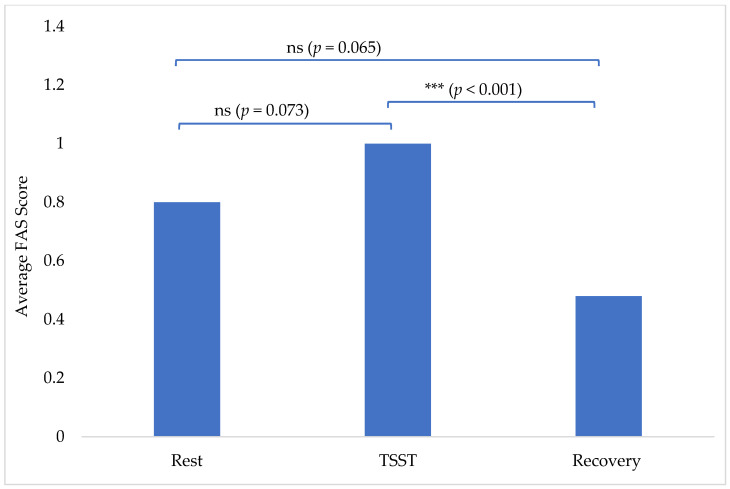
Average FAS score per phase: rest, TSST, and recovery. (ns = no statistical significance and *** = statistical significance).

The average FAS scores insignificantly increased from 0.80 at rest to 1.00 during the TSST (*p* = 0.073). There was then a statistically significant decrease from the TSST (1.00) to 0.48 at recovery (*p* < 0.001; Figure 1). Recovery FAS scores of 0.48 were also insignificantly decreased from the rest scores of 0.80 (*p* = 0.065) (Figure 1). There were no outliers, as assessed by a visual inspection of a boxplot. All variables were normally distributed, as assessed by Shapiro–Wilk’s test (*p* > 0.05).

**Figure 2:**
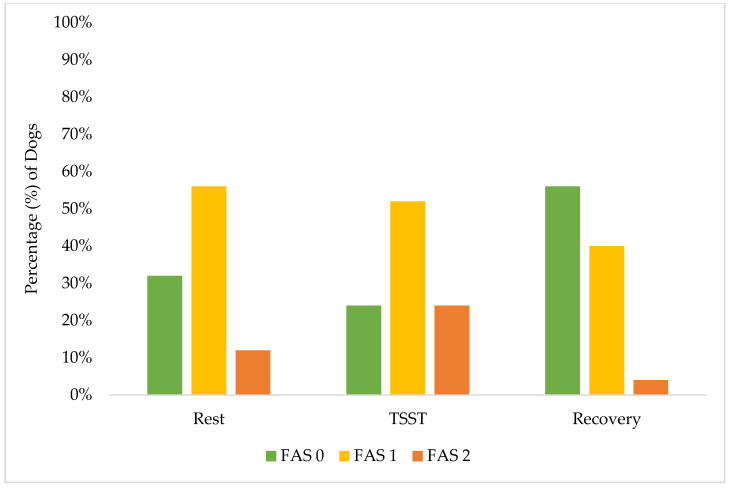
Percentage distribution of FAS score by phase: rest, TSST, and recovery.

**Figure 3:**
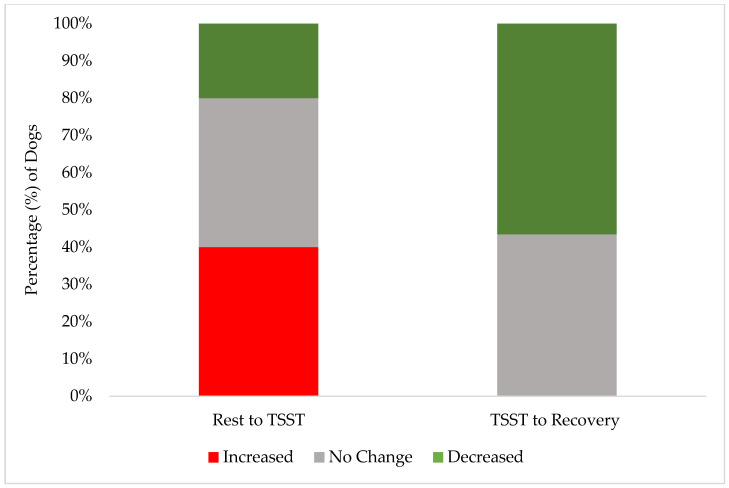
The stacked distribution of FAS score changes between phases: the percentage of dogs whose FAS score increased, decreased, or had no change from the end of the rest phase to during the TSST (“Rest to TSST”); the percentage of individuals whose FAS score increased, decreased, or had no change from the TSST to the end of the recovery phase (“TSST to Recovery”).

These results demonstrate the effectiveness of our screening procedures and study design in maintaining canine welfare throughout the research protocol. While individual dogs showed a variation in stress responses, with 40% (10 of 25) exhibiting increased FAS scores from rest to the TSST, their peak stress remained within acceptable limits (Yellow Zone). A greater percentage of individual dogs (15 of 25, 60%) had no change or a decrease in their FAS score from rest to the TSST. Only 24% (6 of 25) reached an FAS score of two (Yellow Zone) during the most challenging TSST phase, compared to 12% (3 of 25) during rest and only 4% (1 of 25) during recovery. This one individual dog also did not increase to an FAS score of two during recovery but rather remained static from an FAS score of two during the TSST. Notably, the recovery response from the dogs was robust: no dogs showed increased FAS scores from the TSST to recovery, while 52% (13 of 25) demonstrated decreased FAS scores during this transition. These findings are better demonstrated in Figure 2 and Figure 3. By the final recovery phase, 96% of dogs (25 of 26) achieved FAS scores of zero or one (Green Zone), indicating a relaxed or minimally stressed state. No dogs required additional time in the rest or recovery phases to de-escalate their FAS score. These findings validate that our screening process successfully identified dogs capable of participating without severe distress, while our study design and monitoring procedures effectively prevented an unreasonable stress escalation and ensured a rapid return to baseline levels.

We were unable to analyze canine salivary samples for several reasons. First, due to our choice not to continue attempting to collect saliva from dogs who continuously turned or moved away from the swabs (displayed FAS score of two or above). In addition, several dogs had dry mouths with an insufficient sample quantity. We only gathered sufficient samples from 10 dogs. Three of those samples had cortisol concentrations below detectable limits as reported by the lab, making the results unreliable. The remaining seven dogs’ samples were far fewer than necessary to reach statistical power.

## 4. Discussion

This study demonstrates that systematic planning for and monitoring of canine welfare in HCI research is both feasible and essential. The primary study design for Gandenberger et al., 2024 [16] focused on evaluating the physiological and psychological effects of having a pet dog present during an acute stressor on human participants [16]. Concurrently, we implemented comprehensive measures to minimize stress on participating dogs, guided by best practices from AAIs, veterinary medicine, and animal welfare. We collected canine FAS scores and salivary cortisol samples, when possible, to objectively track dogs’ stress levels, allowing us to evaluate our interventions’ effectiveness at achieving low baseline stress levels and preventing unreasonable escalations during the experiment.

While HCI research typically centers human participants, the welfare of canine participants is equally critical from an ethical standpoint. The systematic collection of canine stress data in this study creates an opportunity to analyze and report on canine welfare considerations; an area often overlooked in HCI research. In the following sections, we examine each component of our study protocol, identifying successful approaches alongside areas for improvement in future research. We conclude by proposing preliminary guidelines to help researchers develop study designs that systematically protect the welfare of canine participants in HCI research.

### 4.1. Preparation and Screening for Canine Participants

In preparation for this study, we assessed the study environment to identify and mitigate potential stressors for participating dogs (as described in Section 2.4). Before being accepted into the study, participants completed a custom-designed questionnaire to assess their dog’s suitability (Supplemental Figure S1). While we cannot confirm why nearly one-quarter of individuals who expressed an interest in the study did not follow up after being provided further details and screening questions, some of them likely considered their dogs to be a poor fit for the experiment. Of those who did reply, 12.5% explicitly declined due to concerns about their dog’s suitability. Additionally, only 2 of 26 participants (7.7%) withdrew due to distressed behavior (FAS score of three) from their dog, and the highest recorded FAS score in the final data set was two, the lowest score in the Yellow Zone. No dogs in the study displayed FAS scores of four or five (Red Zone). These outcomes suggest we successfully balanced canine welfare and safety considerations with participant recruitment needs, as evidenced by the low withdrawal rate and low FAS scores observed throughout the study.

Since the Gandenberger et al., 2024 study [16], Wilkins, 2024 [30] validated the C-BARQ^(s)^, a shortened 42-question version of the widely accepted canine temperament assessment that reduces completion time to under 10 min while maintaining validity. For volunteer-based studies like Gandenberger, 2024 [16], the C-BARQ^(s)^ offers advantages in efficiency, standardization, comprehensive assessment, and reduced specialist screening requirements. While effective for assessing aggression and obvious stress, researchers could enhance it by incorporating verbal descriptions of subtle signs of anxiety identified by Mariti et al., 2012 [31], whose research found that dog guardians often recognize overt signs of canine stress (such as whining, trembling, or panting) but overlook subtle cues (such as yawning, lip licking, or turning head away), with women more likely than men to rate their dogs as moderately stressed. Given the overrepresentation of women in Gandenberger et al., 2024 [16], participants may have been more attuned to subtle stress signals displayed by their pet dogs when completing the screening questionnaire.

### 4.2. Arrival at the Study Site, Study Intake, and Informed Consent

After obtaining informed consent, participants provided essential data about their dogs (Table 1), a step that research suggests is frequently neglected. According to Horowitz, 2021 [32], dogs in HCI studies are often treated as objects of investigation rather than true participants. Griffin, 2019 [33] further notes that most studies fail to report even fundamental canine demographics, such as age, sex, and reproductive status, highlighting the importance of our comprehensive documentation approach. Dog behaviors are significantly influenced by these fundamental factors, as well as by their weight, origin (shelter, breeder, or rescue), household composition (presence of other animals or children), and guardian’s experience with dogs [34,35,36,37]. Collecting comprehensive canine demographic data not only respects individual dog welfare but also enables researchers to identify meaningful patterns in this complex field. For instance, the data gathered in this study could later be analyzed to explore potential correlations between specific canine data (such as guardianship duration) and specific participant data (such as the strength of the acute stress response). While HCI research routinely collects detailed human demographic information, thorough documentation about canine partners remains just as crucial for understanding the nuanced dynamics of human–dog relationships.

In addition to collecting canine data, ensuring participants understand their right to withdraw from the study if their dog shows distress is essential for canine welfare in HCI research. While human participants in federally funded studies are protected under the Code of Federal Regulations (45 CFR §46.116(a)(8)) [38], requiring IRBs to enforce withdrawal options during consent, research by Gordon et al., 2006 [39] revealed that none of the 114 consent forms examined provided explicit withdrawal instructions. To address this gap, our study’s consent form explicitly stated “You are also free to end your participation in the study at any time, for any reason, without penalty, including if you feel that your dog becomes too distressed for you to ethically continue participating. You will still receive your gift card incentive even if you end your participation early or decline to respond to any specific questions”. To make withdrawal even clearer, future consent forms should include explicit instructions on how to withdraw, such as “Tell any research facilitator that you’d like to withdraw. You can provide a reason, or you can simply say you’d like to withdraw”. As HCI research continues to expand, future canine welfare guidelines should specifically require that informed consent procedures clearly communicate participants’ right and responsibility to withdraw if they observe any signs of medical or behavioral distress in their dogs, as well as explicit instructions on how to withdraw.

### 4.3. Rest and Recovery Periods

The off-leash exploration time during these periods enabled the dogs to investigate the new environment through sniffing; a critical behavior shown to significantly reduce stress levels in clinical settings [22,23,24,28]. This olfactory investigation serves as a natural calming mechanism by providing essential environmental information and establishing familiarity with novel spaces [40]. The freedom of movement also facilitates exploratory behaviors and autonomic social interactions, which research demonstrates are crucial for physical and emotional welfare while mitigating anxiety and frustration [27]. By combining comfortable resting accommodations with the freedom to move away from human interaction, even from their guardians, the study design minimized the potential stress for the dogs. For future studies, we recommend encouraging participants to bring blankets from home to place over the provided dog beds, as familiar scents have been shown to promote safety, comfort, and calm in novel environments [21,24].

In humans, the waiting or rest period is critical to the TSST protocol because it reduces the psychological and physiological variability related to the travel to the research facility, as well as the anticipatory anxiety or stress related to being in a new and unknown environment [41,42,43]. Similarly, Hernander (2008) [23] found that in a veterinary setting, more than 60% of dogs showed a decrease in stress levels during their time in the waiting room (even in the face of unfamiliar dogs, unfamiliar people, excessive noise, and activity), suggesting that dogs also emotionally regulate over time, after the stress of travel and entering a novel environment. When collecting physiological data that can be affected by stress in companion animals, current best practices suggest waiting 8–10 min to allow the animal to acclimate to the hospital [44].

### 4.4. Stress Test and Observational Data Collection

During the TSST the participants were led into a conference room with a panel of three panelists wearing white lab coats. The panelists were instructed not to give verbal or non-verbal feedback. These aspects of the test were designed to maximize stress on the human participants [41]. However, as research on the complicated relationship between companion dogs and the human stress response grows, it is important to consider how these experimental details impact the canine participants. The “white coat effect” (WCE) is a commonly accepted term in human and veterinary medicine to describe a patient experiencing stress in anticipation of a medical visit [45]. The white lab coats are a purposeful component of the TSST to increase the stress response during the experiment; however, it should be noted that white coats or similar visual indicators of impending medical care have been shown to impact dogs as well. A study examining fear-related behaviors of dogs in the veterinary clinic found that half of the dogs displayed fearful behavior simply walking into the veterinary clinic [46].

Although the average FAS score insignificantly increased from rest (0.80) to the TSST (1.0), the only statistically significant finding in the canine FAS data is the decrease from the TSST (1.0) to recovery (0.48). It is important to recognize that these averages are still very low FAS scores, indicating on average Green Zone behaviors (correlating to relaxed or only very mild anxiety), compared to a statistically significant near doubling in the self-reported State-Trait Anxiety Inventory for Adults (STAI-AD) in all participants (both dog and no dog groups) immediately after the TSST in Gandenberger et al., 2024 [16]. Additionally, all participants (both dog and no dog groups) demonstrated a significant effect of the TSST on heart rates and plasma cortisol levels as well, indicating the TSST successfully elicited a stress response in all participants [16]. Although correlation analyses between human and canine stress responses were not within the scope of this study, we agree that further research should be focused on bidirectional fear transmission between humans and dogs. Future researchers interested in this topic should recognize that it is important to collect more than one stress marker (such as FAS, heart rate, and successful salivary cortisol samples), as was discussed and collected for the human participants in Gandenberger et al., 2024 [16]. However, as will be discussed in Section 4.5, much of this data in canines is still unreliable and limited, making this topic that much more difficult to study.

Only 40% of dogs showed increased FAS scores from rest to the TSST, with 52% decreasing from the TSST to recovery, (Figure 3). While this could be explained by a multitude of factors, it is interesting to note that these numbers strongly mirror the findings in Hernander, 2008 [23], which showed dogs’ fear scores collected as they entered a veterinary clinic decreased after being given the ability to rest and settle for 10 to 20 min in the waiting room. However, the fear scores increased again when moving from the waiting room onto a scale to collect weight (the scale was located at the entrance of a long and narrow hallway, which led into the exam room, where a veterinary exam would commence). These correlations suggests that in our secondary analysis of participant dog FAS scores throughout the primary experiment, the WCE—decreased fear scores when allowed time to rest and settle in a novel environment, followed by increased stress scores upon entering a new room that might predict medical handling (a room of panelists wearing white lab coats)—should be heavily considered as a possible cause for the increased percentage of dogs who had an increased FAS score from rest to the TSST.

In addition, both this study and the Hernander, 2008 [23] study showed a very similar percentage of dogs whose fear score or FAS score remained unchanged for the duration of the evaluation, with this study finding 6 of 25 (24%) of the dogs had a completely constant FAS score throughout the entire experiment and Hernander, 2008 [23] finding 25.7% of dogs had a completely unchanged fear score throughout the evaluation. It is also interesting to note that, of the six dogs who had a completely unchanged FAS score at each data collection point, four dogs (67% of the unchanged FAS score group and 16% of the total group) had an FAS score of zero throughout the experiment, and two dogs (33.3% of the unchanged FAS score group and 8% of the total group) had an FAS score of one throughout the experiment (all Green Zone). In addition, any dog that had an FAS score of zero at any recorded time in the experiment never displayed an FAS score higher than one. This might suggest that dogs with lower stress scores overall might have a higher resilience to stressors in general. Additional research into this topic, especially as it relates to HCI research, should be further explored in future studies.

It is tempting to attribute the insignificantly increased FAS in the dogs during the TSST to the participants’ significant stress response to the TSST, given research supporting canine social referencing, especially related to their guardians [47,48]. However, in addition to the limited canine stress markers successfully collected in this study (FAS scores only) and the many confounding factors (such as the WCE), Merola, 2014 [48] also suggests that in their daily life, dogs may be more exposed to expressions of happiness than fear from their guardians, and therefore they may have had more chances to associate specific outcomes with positive emotions rather than negative emotions. They found that dogs used their guardians’ expression of happiness, rather than avoiding the expression of fear, to make decisions about what novel box to explore [48]. Data on canine social referencing with humans must be carefully considered for any future research further exploring this topic, especially as related to HCI studies.

The FAS score as the only stress marker collected and analyzed for the dogs at all three data points in this experiment is a limitation. The FAS score being collected by only one researcher is another limitation. This could have been improved upon by having multiple trained individuals collect the FAS scores to assess for inter-rater reliability, or a video recording of the experiment could have been collected to have canine behavior specialists score the FAS, along with the Fear Free-certified study facilitator. However, given the additional cost for these solutions and noting the dogs’ stress response was not part of the research question for the primary study design, this is something that should be further explored in future studies.

The TSST FAS score data point was recorded as the most consistent body language seen while the participant was in front of the panelists. It is important to note that the nuance of the FAS score in this or similar experiments could be better captured by more frequent FAS score data points, such as when entering the rest room, when exiting the rest room, when entering the TSST room, at the start of the participants’ interaction with the panelists, at the end of the participants’ interaction with the panelists, while exiting the TSST room, when entering the recovery room, and when exiting the recovery room. This would help further assess whether any increased observed stress during the TSST might be related to a mirroring response to the participants’ stress response or might be related to external factors, such as the WCE.

Lastly, it is important to note that while the primary study design included a control group of participants without dogs, the present secondary analysis focuses solely on canine participants with their guardians (the experimental group) since the focus of this study is recognizing, mitigating, and tracking canine stress in HCI research. The lack of a true control and experimental group in this secondary analysis is recognized as a limitation of this study. This limitation means our findings reflect canine welfare outcomes specifically within the context of dogs participating alongside their familiar guardians during a human stress task.

If future studies wish to focus on canine–human social referencing, canine–human mirroring responses, or the bidirectional emotional influence in guardian–dog dyads in HCI research, the guardian and pet dog would be the control group, and experimental groups to consider could include guardians participating with unfamiliar pet dogs (and equally pet dogs participating with unfamiliar handlers) or humans participating with unfamiliar trained therapy dogs. Future research could explore canine welfare outcomes across different handler–dog relationship conditions to better understand how the human–canine relationship affects both human and canine stress responses during research participation and to establish more comprehensive welfare guidelines for various HCI research contexts.

### 4.5. Physical Data Collection (Blood and Saliva Sampling)

During the participant blood collection, the research facilitator took control of the dog’s leash, keeping the dog visible but beyond the participant’s reach—a critical safety measure. Despite the potential for negative reactions from dogs experiencing brief separation or sensing their guardian’s discomfort during blood draws, only one dog needed to withdraw, confirming our effective screening process and successful balance of canine welfare with research requirements.

To further improve canine welfare in future HCI research, we might consider Csoltova et al., 2017 [49]’s findings that owner interaction significantly improves dogs’ stress responses during veterinary examinations. This study showed dogs were less likely to attempt an escape when guardians engaged with them, suggesting a calming effect. Conversely, when guardians remained visible but non-interactive (similar to our blood collection procedure), dogs showed an increased motivation to escape and seek owner proximity. While physical contact during blood collection is not feasible or safe, participants could be encouraged to speak reassuringly to their dogs during these brief separations to promote a sense of calm.

In our study, we were unsuccessful in collecting a sufficient number of canine salivary cortisol samples to allow for an analysis that reached statistical power. Unfortunately, the current research does not suggest a simple, reliable, or easy solution to this limitation. There is no currently accepted method of increasing salivary production for collection and analysis in dogs that does not potentially alter the results. Dreschel & Granger, 2009 [50] found that beef-flavored ropes were found to introduce an unpredictable variability to cortisol concentration measurements. In addition, they found that higher levels of citric acid falsely elevated salivary cortisol results [50]. Wiping a dog’s mouth with a citric acid-soaked cotton ball can increase salivary production without falsely elevating the cortisol results; however, dogs resisted having their mouths wiped with a cotton ball [50]. Given the difficulty of simply utilizing the salivary collection swab in this study, this would likely not be a useful technique, though it could be considered in studies where dogs could receive more training and counterconditioning prior to the sample collection. One research-supported consideration to improve the success of salivary cortisol data collection is to individually train dogs to use a sampling device in the mouth 5 days before the experiment starts [51]. The classical conditioning of the salivary swab with treat rewards for 5 to 7 days before the study might also create a conditioned salivary reflex to the swab insertion (without the need for the treat reward during the study), although this concept is only theorized by the authors and has not been tested. However, the time, participant commitment, and participant reliability, as well as the cost associated with creating a reliable training video or other attempts at training, could be limiting in many studies.

It is also important to note that multiple current studies warn about the poor reliability of canine salivary cortisol as a stand-alone marker of stress. The difficulties in the collection of an adequate sample quantity (as well as lack of reliable methods to increase salivary production without interfering with results), lack of standardized sampling methods, intraindividual and interindividual variability, environmental factors (such as living environment and recent experiences), lack of a correlation between signs of stress and cortisol levels, lack of established reference ranges, variability in results related to the spay/neuter status and sex, variability related to health status, social context of the stressor, and circadian alternation are just a few of the myriad possible complications associated with the canine salivary cortisol marker [50,52,53,54,55].

Additionally, data show a lag in the peak cortisol response to a stressor of 16 to 20 min [52], making the correlation of the stressor and cortisol response in research potentially difficult. Since the TSST only lasted approximately 13 min [16], and the study team accounted for this lag by collecting the participant blood samples first, then participant salivary samples, and lastly the canine salivary samples. The rest phase lasted 30 min and would have accounted for any lag in stress from travel and study arrival. The recovery phase was 15 min, but with additional time for gathering supplies and guiding participants on salivary sample collections, this likely accounted for the documented lag.

Finally, it would be useful to standardize when to discontinue attempts at salivary swab collections to increase the chance of success and reduce individual research facilitator bias on when to discontinue attempts. The recommended standardization could be outlined based on the Fear Free Canine FAS sedation–pain algorithm [56], where if an FAS score of zero to one is seen, the continuation of the collection is allowed. If an FAS score from two to three (fidgeting, difficulty settling, and head turning away) is seen for more than 3 s, the dog is given a break, and if the FAS score returns to zero to one the attempt at collection is resumed; however if the dog returns to an FAS score of two to three, the attempt would be discontinued and the dog would be withdrawn from the study. If an FAS score of four or five is seen, the attempt is immediately discontinued, and the dog is withdrawn from the study.

### 4.6. Additional Methods to Track Canine Stress: Considerations

Our study design utilized canine behavioral observations (FAS score) and salivary cortisol levels to track stress, with a failure of success in analyzing the salivary cortisol data, limiting our stress tracking to three data points (FAS scores at rest, TSST, and recovery). However, due to the complexity of the canine stress response, an assessment with multiple complementary systems is recommended to develop a more comprehensive understanding of canine stress in HCI research. Below, we discuss additional objective physiological monitoring technologies that exist or show promise for assessing stress in canine participants.

Kooriyama & Ogata, 2021 [57] published an extensive and in-depth study on salivary stress markers in dogs, including cortisol, chromogranin A (CgA), salivary alpha-amylase (sAA), and proinflammatory cytokines. However, most of these measurement techniques remain in developmental stages and require analyte-specific assays and laboratory analysis that require access to specialized equipment and expertise, which can be prohibitive for many research projects [57]. Additionally, there are ongoing challenges in the detection sensitivity and standardization [57]. As previously mentioned, another limitation is proper saliva collection protocols and standardized timing, as levels of these biomarkers can fluctuate throughout the day or in response to stressor exposures, adding further complexity to the research implementation [57].

A more commonly accepted, reliable stress data point to monitor is the canine heart rate (HR). Historically, reliable HR data collection required invasive and expensive equipment, such as a Holter monitor. However, advances in canine activity monitoring technology now allow simple and reliable options. For example, the PetPace™ collar allows the collection of not only the HR but also other data, such as activity, body position, respiratory rate, and body temperature [58]. It is important to note that some dogs may find the collars irritating or uncomfortable, potentially causing behavioral changes that affect measurements and raising welfare concerns, although the risk of this is likely low compared to the potential benefit of the data. Physiological data accuracy presents another challenge, as vital sign measurements can be influenced by activity, sounds such as barking, or a poor collar fit, potentially yielding unreliable results without the careful attention to proper device positioning and data validation [58]. More importantly, for our intended purposes in monitoring canine stress during HCI studies, the PetPace collar was found to have no statistically significant difference in the mean HR when compared to a Holter-ECG; the gold standard in measuring canine HR [58]. However, commercial activity monitors like PetPace are relatively expensive, requiring an investment in both hardware and ongoing subscription services, which may limit accessibility for some research projects.

## 5. Conclusions

There is currently a lack of institutional or research guidance on the ethical incorporation of canines into HCI research, especially volunteer participant pet dogs. It is imperative that future researchers carefully develop, share, and report on the successes and failures of their protocols so that standardized guidelines can be created. If care is not taken, canine welfare may be compromised, participant and researcher safety could be endangered, and experiments may be unable to continue responsibly.

Our research demonstrates how to successfully design a study that plans for the welfare considerations and stress monitoring of canine participants. While our protocols were designed for a specific study and within a specific context, the broader principles are outlined as “Proposed Guidelines for HCI Canine Welfare” in Table 2. We hope that other researchers who wish to study HCIs will use this information to design studies that incorporate care for all living beings involved. Such a multispecies-focused and ethical approach will lead to more humane and ethically sound research practices, fostering positive outcomes for both canine participants and the humans they interact with, as well as enabling researchers to better understand the nuanced and complicated dynamics of human–canine relationships.

## Figures and Tables

**Table 1:** Canine demographics collected for 26 pet dogs. We collected dog age, length of guardianship (ownership) between the participant and their pet dog, sex, spay/neuter status, and breed.

Demographic	Results	n (%)
Dog Age	0–1 years	3 (11.5%)
	2–3 years	5 (19.2%)
	4–5 years	3 (11.5%)
	6–7 years	8 (30.8%)
	8–10 years	4 (15.4%)
	11–15 years	3 (11.5%)
	15+ years	0 (0%)
Length of Ownership	0–1 years	4 (15.4%)
	2–3 years	7 (26.9%)
	4–5 years	4 (15.4%)
	6–7 years	5 (19.2%)
	8–10 years	4 (15.4%)
	11–15 years	2 (7.7%)
	15+ years	0 (0%)
Sex	Male	12 (46.2%)
	Female	14 (53.8%)
Spayed/Neutered	Yes	24 (92.3%)
	No	2 (7.7%)
Breed (owner-reported)	Alaskan Malamute	1
	Australian Cattle Dog	1
	Australian Shepherd	1
	Chihuahua	1
	English Lab	1
	English Springer Spaniel	1
	German Shepherd	2
	Goldendoodle	3
	Havanese	1
	Persian Mastiff	1
	Siberian Husky	1
	Standard Poodle	1
	Toy Poodle	1
	Other mixed breed	10

One experiment ended before these data were collected.

**Table 2:** Proposed guidelines for HCI canine welfare.

Preparation and Screening for Canine Participation
During participant recruitment, enough detail of the study needs to be provided to allow potential participants to determine if they and their dog are good candidates for the study. Researchers should walk through the entire study design and evaluate all aspects from the canine perspective. When possible, known stressors, such as loud noises, strong odors, the risk of pheromones from nearby stressed dogs, slippery surfaces, and bright lights, should be mitigated [8,21,22,23,24,25,26]. Participants must complete a screening questionnaire about their dogs’ behaviors and temperament to allow researchers to assess canine compatibility with the study conditions. Consider the C-BARQ^(s)^ for efficiency, standardization, comprehensive assessment, and reduced specialist screening requirements [30]. Adopting the C-BARQ^(s)^ would also maintain anonymity regarding the study goals and conditions while still screening for unfit dogs. Where needed, the questionnaire can be modified to include additional pertinent questions, such as those related to canine mobility or potential exposures to dangerous or toxic items, as well verbal descriptions of subtle signs of stress seen in dogs [31].
Arrival at the Study Site, Study Intake, Informed Consent
Study facilitators should be properly educated on both assessing canine body language and knowing how to utilize their own body language to avoid appearing threatening and to minimize stress [21,22,24,26,28]. Consider the Fear Free™ Trainer Certification program, as it is the most relevant to reducing stress in dogs utilized in HCI research [20]. If Fear Free releases a certification designed for researchers, prioritize that program. Collect comprehensive canine demographic data. A minimum database should include age, sex, reproductive status, weight, and length of guardianship. When possible, additional data including canine origin, household composition, and guardian experience with dogs should also be included [23,35,36,37]. Ensure informed consent contains verbiage that makes it clear the participant can withdraw at any time, for any reason, without penalty, including if they feel their dog is in any medical or behavioral distress. Consent should also provide explicit instructions on how to withdraw.
Study Environment
The study facility should be optimized for canine comfort wherever able. The study location should include a dog bed, a water bowl, water refills, proper traction on flooring, and an air purifier to decrease aversive scents or scents of other animals; an additional white noise machine can be considered [6,22,25,26]. If able, encourage the participants to bring a blanket from home to place over the provided dog bed, as familiar scents have been shown to promote safety, comfort, and calm in novel environments [21,24]. If a separate room with a door is available, allow dogs to have off-leash time. This enables de-stressing through sniffing and exploring, autonomic social interactions, and the opportunity to move away from human interactions if necessary. This, in turn, promotes physical and emotional welfare and mitigates anxiety and frustration [22,23,24,27,28,40]. If not built into the study protocol, a rest or waiting period is critical to allow for emotional regulation after traveling and acclimating to a novel environment. A minimum of 8 to 10 min is recommended based on best practices for collecting physiological data affected by stress in the veterinary clinic [44].
Canine Stressor(s)
Consider the “white coat effect” when designing the study [45]. Any acceptable increase in dogs’ stress response should be pre-determined during the study design so that study facilitators know when to exclude a canine participant. If using the Fear Free Canine Spectrum Fear, Anxiety, and Stress (FAS) scale [18], the recommended standardization could be outlined based on the Fear Free Canine FAS sedation–pain algorithm [56]. This would allow dogs who show an FAS score above a 1 for longer than 3 s time to recover (if allowed without invalidating study results). If the dog is unable to return to an FAS score below 2, or a break cannot be administered, the dog should be withdrawn for best welfare practices.
Observational Data Collection
If using behavioral observations, such as FAS scores, as a canine stress monitoring datapoint, this should be collected as frequently as possible. Minimum data points should include the entrance and exit to any new location, as well as the start and finish of any applied stressor.
Physical Data Collection
If the dog must be physically distanced from the guardian at any time, consider encouraging the guardian to speak reassuringly to their dog to promote calm [49]. If collecting canine salivary samples, consider detailed training videos guiding participants on how to counter-condition their dog to the collection method, aiming to begin training 5 days before the experiment starts [51]. Classical conditioning to the salivary swab with treat rewards for 5 to 7 days before the study might also create a conditioned salivary reflex to the swab insertion (without the need for the treat reward during the study). Be aware that research shows a lag in peak salivary cortisol in response to a stressor of 16 to 20 min [52]. Standardize when to discontinue attempts at any physical data collection. The current recommended standardization is outlined based on the Fear Free Canine FAS sedation–pain algorithm [56] where if an FAS score of 0 to 1 is seen, the continuation of the collection is allowed. If an FAS score of 2 to 3 (fidgeting, difficulty settling, head turning away, avoidance) is seen for more than 3 s, the dog is given a break, and if the FAS score returns to 0 to 1 the attempt at the collection is resumed; however, if the dog returns to an FAS score of 2 to 3 the attempt would be discontinued. If an FAS score of 4 or 5 is seen, the attempt is immediately discontinued.
Additional Methods to Track Canine Stress: Considerations
Track current research on salivary stress markers in dogs, including cortisol, CgA, sAA, and proinflammatory cytokines [57]. Consider HR tracking with a canine activity monitor, such as the PetPace™ Collar [58].

## Data Availability

To protect participant privacy, the data collected in this study cannot be shared publicly. Upon reasonable request, the data may be made available by the authors.

## Supplementary Materials

The following supporting information can be downloaded at: https://www.mdpi.com/article/10.3390/ani15111665/s1, Figure S1: The custom screening questionnaire, developed by the study team, was sent to anyone who expressed interest in the study.

## Abbreviations

The following abbreviations are used in this manuscript:

HCIs	Human–Canine Interactions
HAIs	Human–Animal Interactions
AAIs	Animal-Assisted Interventions
AAT	Animal-Assisted Therapy
AAE	Animal-Assisted Education
AAAs	Animal-Assisted Activities
IRBs	Institutional Review Boards
IACUCs	Institutional Animal Care and Use Committees
ARRRIVE	Animal Research: Reporting of In Vivo Experiments
LEAD	Lincoln Education Assistance with Dogs
VCSTs	Veterinary clinic studies committees
TSST	Trier Social Stress Test
CGC	Canine Good Citizen
CPDT-KA	Certified Professional Dog Trainer, Knowledge-Assessed
CDBC	Certified Dog Behavior Consultant
IAABC	International Association of Animal Behavior Consultant
FAS	Fear, Anxiety, and Stress
ANOVA	Analysis of Variance
C-BARQ^(s)^	Canine Behavioral Assessment and Research Questionnaire
CFR	Code of Federal Regulations
WCE	White coat effect
STAI-AD	State-Trait Anxiety Inventory for Adults
CgA	Chromogranin A
sAA	Salivary alpha-amylase
HR	Heart rate
