# Supplementary material for: Recognizing and Mitigating Canine Stress in Human–Canine Interaction Research: Proposed Guidelines
Source: Animals (Basel). 2025 Jun 5;15(11):1665. doi: 10.3390/ani15111665 (PMC12153650; doi:10.3390/ani15111665)
Supplement: Supplementary file 1 [file animals-15-01665-s001.zip › animals-3637834-supplementary.pdf]

Supplemental Figure S1. The custom screening questionnaire, developed by the study team, was sent to anyone who expressed interest in the study.

<u>Participant Screening Questionnaire</u>

1. Do you have any experience or training in evaluating dog stress signals? If so, what?
2. How does your dog act when they're stressed? What helps them calm down?
3. Has your dog shown any signs of aggression towards other people or dogs? If so, please explain.

**Select Yes/No/Maybe if you expect your dog to be comfortable in the following scenarios. Participation in this study requires confirming <u>ALL</u> of the below. Please speak to the study facilitator if you cannot confirm all of the examples listed.**			
Taking elevators or stairs up to the 5th floor offices.	YES	NO	MAYBE
Being around several unknown adults—potentially up to 4–5 at a time—most of whom will not greet or interact with the dog.	YES	NO	MAYBE
Leash being held by an unknown adult for several minutes at a time, at three different times, while the human's blood is being drawn. The dog may be in sight of their owner or just around the corner, depending on what the dog seems to prefer.	YES	NO	MAYBE
Being in a laboratory environment that may have strange smells. There shouldn't be anything dangerous on the floors, but it's important that the dog does not lick or eat anything they might encounter.	YES	NO	MAYBE
Being with their owner while the owner is temporarily distressed. The stress may last 15–20 min, or more.	YES	NO	MAYBE
The dog should be leashed at all times except when the dog is alone with the owner.	YES	NO	MAYBE
In general, the dog should be responsive to their owner and comfortable in novel environments.	YES	NO	MAYBE
**We do not expect the dog to encounter any other dogs while they're with us. However, we cannot guarantee this.**			
